# Protocol for a murine transient middle cerebral artery occlusion model with local intra-arterial drug delivery

**DOI:** 10.1016/j.xpro.2026.104618

**Published:** 2026-06-08

**Authors:** Jordan J. Lee, Michael P. Murphy, Thomas Krieg

**Affiliations:** 1 Department of Medicine, University of Cambridge, Addenbrookes Hospital, Hills Road, Cambridge CB2 0QQ, UK; 2 MRC Mitochondrial Biology Unit, University of Cambridge, Hills Road, Cambridge CB2 0XY, UK

**Keywords:** Metabolism, Model Organisms, Neuroscience

## Abstract

Here, we present a protocol for a murine transient middle cerebral artery occlusion (tMCAO) model incorporating an intra-arterial catheter for local drug delivery during reperfusion. We describe steps for pre-operative preparation, surgical procedure, and post-operative care. We detail procedures for the insertion of a microcatheter for targeted drug delivery during reperfusion to mimic clinical mechanical thrombectomy. This approach enables investigation of adjunct therapies for ischemia-reperfusion injury.

For complete details on the use and execution of this protocol, please refer to Lee et al.^1^

## Highlights

• Murine tMCAO model simulating mechanical thrombectomy with local drug delivery
• Instructions for creating an intra-arterial microcatheter
• Insertion of intra-arterial microcatheter during transient occlusion

## Abstract (editor highlights)

Publisher’s note: Undertaking any experimental protocol requires adherence to local institutional guidelines for laboratory safety and ethics.

## Graphical abstract

**Figure:**
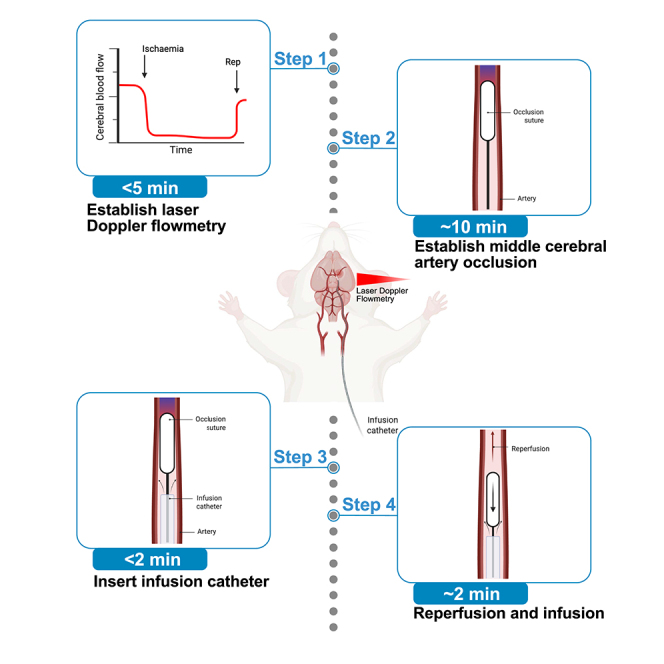

## Before you begin

### Innovation

The protocol below describes an adapted murine endovascular filament transient middle cerebral artery occlusion (tMCAO) model incorporating an intra-arterial catheter. We have used this protocol to investigate acidified disodium malonate as an adjunct therapy to mechanical thrombectomy to minimize ischemia-reperfusion injury (IRI).^1^ The significance of this model is the integration of an infusion catheter with the endovascular filament, enabling local drug administration upon reperfusion, a critical feature to investigate stroke IRI *in vivo* and the efficacy of any potential drugs that target it. Nevertheless, there are potential applications of this method that go beyond IRI, for instance, local administration of hyperpolarized compounds for MRI or radiotracers for PET-CT studies. This protocol is an adaptation of the Koizumi tMCAO method and is, therefore best suited for experienced stroke researchers.

### Institutional permissions (if applicable)

All animal procedures must be carried out in accordance with relevant national and institutional guidelines. The procedures in this protocol were reviewed by the University of Cambridge Animal Welfare Ethical Review Board and approved under Project License PP4344323.

Humane endpoint criteria must be prospectively defined and approved in accordance with local regulations and ethical oversight before study initiation.

### Preparation

**Timing: 30 min**

1. Prepare a sterilized and aseptic workspace.
2. Create an infusion catheter by inserting a thin polyimide catheter through a needle tip and glueing it in place (see Figure 1). Once the glue has set, attach the needle to a 1 mL syringe and flush the catheter with water to ensure that there is no leakage.

**Figure 1:**
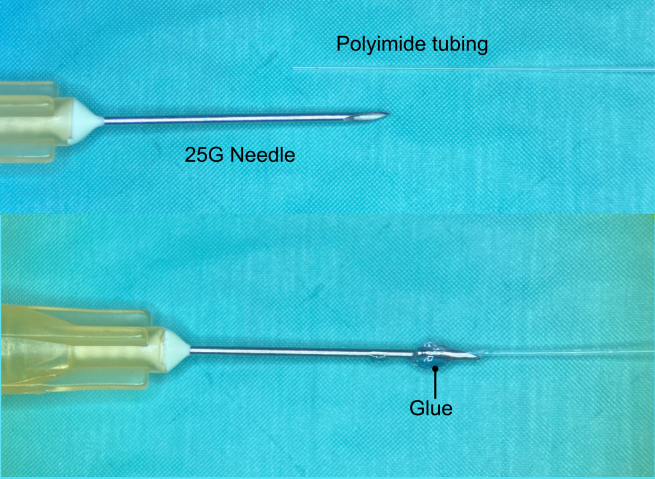
Creating the infusion catheter
3. Sterilize the catheter using 70% ethanol.
4. Set up the operating area and equipment and prepare all necessary surgical tools. Ensure that all surfaces and tools are sterilized.

## Key resources table

**Table:** 

REAGENT or RESOURCE	SOURCE	IDENTIFIER
**Experimental models: Organisms/strains**		

Male C57BL/6 mice	Charles River Laboratories	Strain code: 027

**Chemicals, peptides, and recombinant proteins**		

Chlorhexidine Gluconate 4% w/v	Hibiscrub	N/A
Isoflurane	Zoetis	N/A
Gluture	Zoetis	N/A
Viscotear Liquid Gel	Bausch + Lomb	N/A
Buprevet (0.3 mg/mL)	Virbac	N/A

**Other**		

Leica 3M Microscope	Leica Microsystems	N/A
Polyimide catheter (ID: 172 ± 12 μm; OD: 216 ± 12 μm)	Zeus PN	Zeus PN: 270903
6-0 Surgical Nylon Monofilament Suture	Ethicon Inc.	N/A
6-0 Coated VICRYL	Ethicon Inc.	N/A
PeriFlux System 5000	Perimed	N/A
TEC 3 Isoflurane Vaporiser	+Mediquip LTD	Model:98723N
Occlusion suture	Doccol	602223PK10RE
TLAT-2LV	Physitemp	https://www.physitemp.com/animal-temperature-control_p130
PUMP 11 Elite	Harvard Apparatus	Item No: 70-4504
Needle 25G (0.5 × 16 mm)	BD Microlance 3	N/A
3M Micropore (Tape)	3M	N/A

## Step-by-step method details

### Transient middle cerebral artery occlusion procedure

**Timing: 110 min total per mouse: 10 min for the pre-operative preparation, 5 min for establishing LDF monitoring, 15 min for filament insertion, 30 min for the occlusion time, and 10 min for reperfusion + suturing.**

### Pre-operative preparation

**Timing: 10 min**

This section describes the pre-operative preparation for mice to undergo the tMCAO surgery.

1. Weigh the animal and transfer it to an anesthesia induction box. Anesthetize the animal at 5 (v/v) % isoflurane delivered with 1.5 L O_2_/min.
2. Once the animal is anesthetized (check paw pinch and ocular reflex), transfer the animal to a heat plate and insert a rectal thermometer to monitor and maintain body temperature at 37 °C.

***Note:*** No other physiological monitoring e.g. blood pressure, blood gas analysis was performed.

3. Maintain anesthesia with 1.5% isoflurane delivered by a face mask.
4. Administer 100 μl buprenorphine (dilute Buprevet 0.3 mg/mL 1:10 in sterilized water)subcutaneously for analgesia.

***Note:*** The appropriate analgesia may be dependent on your protocol and experimental design.

5. Shave the ventral area of the neck as well as the area between the left eye and ear. The latter is for the attachment of the Doppler probe.
6. Apply eye ointment to avoid eye dryness during operation.
7. Once all necessary pre-operative preparation has been completed, transfer the animal to the operating bench with a heating pad controlled by a rectal probe and anesthesia face mask.

***Note:*** In our set up, the pre-operative bench is separate from the operating bench.

### Cerebral blood flow monitoring with laser Doppler flowmetry probe

**Timing: 5 min**

This section describes the procedure to establish LDF monitoring in animals during tMCAO surgery.

8. Place the animal on its side (sagittal plane) and disinfect the shaved area between the eye and ear with any appropriate disinfectant. After which, make a small incision in the sagittal direction from the ear to the eye.
9. Retract the skin and remove the periosteum. Attach and glue the doppler probe to the left MCA territory on the parietal bone to monitor blood flow of the MCA territory.
10. Reposition the animal to a supine position and fix the paws with surgical tape.
  ***Note:*** Take care in repositioning the animal as this may detach the doppler probe.

### Transient middle cerebral artery occlusion procedure

**Timing: ∼45 mins: 10–15 min for filament insertion + 30 min occlusion time**

This section describes the procedures of tMCAO.

11. Reposition the animal to a supine position and tape its forelegs to the operating surface.
12. Make an incision slightly right of the midline (for left carotid territory) on the neck. Separate the fascia and retract the skin and salivary glands to expose the carotid artery.
13. Once the common carotid artery (CCA), internal carotid artery (ICA), and external carotid artery (ECA) have been identified, carefully remove the surrounding fascia to isolate arteries. Be careful not to damage the vagus nerve during this step.
14. Wrap two 6-0 nylon sutures loosely around the CCA, and a third around the ECA.
15. Tighten the ECA suture and the lower (proximal) CCA suture.

***Note:*** It is recommended to have the proximal suture as low down on the CCA as possible to create space for the infusion catheter.

16. Place a vascular clamp on the ICA and make a small incision on the CCA close to the proximal suture.

***Note:*** Similarly, it is recommended to have the CCA incision as low as possible to create an optimal length between the incision and the vascular clamp.

17. Insert the endovascular filament into the CCA via the incision and secure the filament in place with the top (distal) CCA suture. Note: Endovascular filament specifications: coating length: 2-3 mm, tip diameter: 0.22 mm
18. Remove the vascular clamp on the ICA and gently advance the filament via the ICA towards the MCA. Insertion depth: usually 8-10 mm or a feel of slight resistance and confirmed by changes on LDF.
19. Occlusion of the MCA is confirmed when a sharp and sudden decline in the LDF readout is observed. Animals that do not have a > 70% drop in cerebral blood flow relative to baseline are excluded from the study.

***Note:*** Occlusion/ischemia time will be dependent on your protocol and experimental design.

***Note:*** Anesthesia is maintained throughout the MCAO period.

### Insertion of intra-arterial catheter for local drug delivery

**Timing: 5–10 min (Depends on infusion duration and includes suturing)**

This section describes the detailed procedure for the insertion of an intra-arterial catheter for local drug delivery during reperfusion of the tMCAO model.

**Figure 2:**
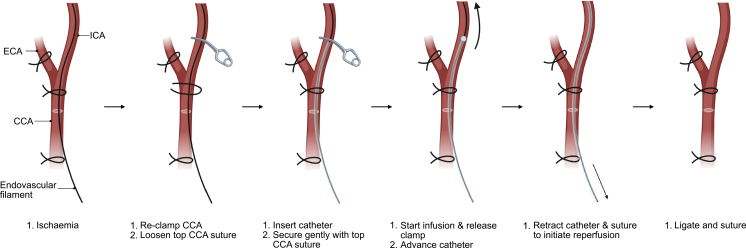
Schematic of protocol with step-by-step instructions

***Note:*** This part of the procedure requires challenging microsurgical techniques and will require patience and perseverance to master. Refer to Methods Video S1 and Figure 2 for detailed surgical procedure.

20. Make sure that the catheter is pre-filled before insertion and place the syringe on an infusion pump.

***Note:*** The catheter has ∼7 μL dead space.

21. Ensure that the catheter is flushed with saline/drug prior to insertion.
22. Approximately 1.5–2 minutes before reperfusion, re-clamp the ICA and loosen the top (distal) CCA suture (see troubleshooting 1).
23. Carefully insert the infusion catheter onto the endovascular filament so that the filament is inside the catheter.
24. Carefully guide the catheter into the CCA and advance it towards the vascular clamp (see troubleshooting 2).

***Note:*** The timing of when to insert the catheter will vary depending on operator experience and experimental design.

25. Once the catheter is in the CCA, gently tighten the top distal CCA suture to secure the catheter and to prevent backflow (see troubleshooting 3).

***Note:*** Be careful not to over tighten the suture otherwise the infusion will not work.

26. Start the infusion with a syringe pump and immediate remove the clamp.
27. Gently advance the catheter (guided by the endovascular filament) towards the MCA (see troubleshooting 4 and Methods Video S1).

***Note:*** The insertion depth of the endovascular filament is usually 8–10 mm, therefore the maximum the catheter can be advanced is 6–8 mm (depends on the coating length of the filament). The infusion volume and rate in the video is 25 μl at 25 μl/min.

28. Then gently retract both catheter and filament to initiate reperfusion, confirmed by LDF readings.

***Note:*** The filament cannot be retracted independently as it is inside the catheter (see troubleshooting 5).

29. Once the infusion has been completed. Fully retract both the infusion catheter and endovascular filament and permanently ligate the CCA by tying both CCA sutures. The ECA suture can be loosened and removed.

***Note:*** Infusion volume and rates will differ depending on experimental design. New researchers may refer to Table 1 which summarizes the infusion regimens that were tested.

30. Remove the retractors and suture the incision with 6-0 sutures. Apply disinfectant when completed.

**Table 1:** Tested infusion regimen

Volume (μl)	Infusion rate (μl/min)	Total infusion time (min)
50	12.5	4
50	25	2
25	25	1
31. Remove the Doppler probe from the skull and close the wound with tissue adhesive or sutures. Apply disinfectant when dried.

***Note:*** Tissue adhesive may be more appropriate than needle sutures. Saline (0.9%) or dextrose saline may also be administered subcutaneously at this step for hydration.

***Note:*** The infusion catheter can be reused (depending on study design) provided that it is cleaned and sterilized after use. This can be done by flushing the catheter with 70 % ethanol. However, if the study requires high aseptic standards such as recovery or immunological studies, it is recommended that a new catheter be used.

Methods Video S1. Insertion of an intra-arterial catheter for local drug delivery, related to step 24

### Post-operative care

**Timing: Min. 1 h (Depends on study design and animal care guidelines)**

This section describes the post-operative care for animals following tMCAO surgery.

32. Turn off anesthesia and transfer the animal to a separate post-operative cage with appropriate bedding, easily accessible wet mash (or other soft foods) and water and place in a warm cabinet (25 °C). Administer the appropriate analgesia if required.
  ***Note:*** Post-operative care procedures will depend according to local regulations and animal licensing.
33. Periodically monitor the animals to assess recovery. If conditions exceed pre-defined parameters, perform appropriate euthanasia.
34. Monitor and weigh the animals daily. Hydration support (e.g., subcutaneous saline) is recommended to aid recovery of animals.
35. Euthanize the animal at a pre-defined endpoint.

## Expected outcomes

Animals subjected to tMCAO will generally display acute changes in behavior and general health. Mice can experience over 20% reduction in weight relative to baseline and display varying degrees of paralysis and functional deficits such as circling, head tilt, forelimb flexion etc. Neurological scores such as the modified neurological severity score (mNSS) can be used to assess and monitor animals. Furthermore, moderate infarction in the striatum and cortex is generally observed (depends on ischemic duration). Mice with no infarct or subarachnoid hemorrhage, determined by blood clots or blood diffusion around the circle of willis at the base of the skull should be excluded from further analysis.

## Limitations

The tMCAO model is a complex surgical procedure and requires significant training to ensure robust and reproducible results. The adaptation of this model to incorporate an intra-arterial catheter for local drug delivery during reperfusion further increases the model’s complexity. As such, even experienced tMCAO surgeons will require significant practice to master. Nonetheless, it should take ∼20 attempts to become familiar with the protocol. Additionally, insertion of the intra-arterial catheter may induce vessel damage and lead to microthrombi or hemorrhage, to reduce the risk of vessel damage it is recommended not to advance the infusion catheter as deep as possible.

This protocol is adapted from the Koizumi’s method of tMCAO and therefore describes permanent ligation of the CCA which may have implications to long-term perfusion, infarct evolution, as well as behavioral outcomes. Limitations of the Koizumi’s method has been extensively discussed in the literature.^2^

## Troubleshooting

### Problem 1

Early reperfusion during re-clamping of the ICA for catheter insertion (Step 26).

### Potential solution

• When re-clamping the ICA in this step, it is normal to observe noise on the LDF readings. This should normalize back to the ischemic reading. However, there is a chance that re-clamping the ICA will induce reperfusion, and therefore the animal will have to be excluded. When re-clamping the ICA, try to make sure the clamp does not retract or pull back the endovascular filament.

### Problem 2

Difficulty inserting the catheter into the CCA (Step 27).

### Potential solution

• Using forceps, gently advance the catheter over the filament into the CCA. Make sure to move the catheter independently of the filament, and also not to pinch too hard as this will deform the catheter.
• Ensure that the length between the ICA clamp and top CCA incision is sufficient to accommodate the catheter.

### Problem 3

Backflow during infusion (Step 28).

### Potential solution

• Ensure that the top CCA suture is secured and gently tightened. Be careful not to over tighten as this will prevent infusion flow.
• If there is blood backflow into the catheter, make sure to start infusion prior to releasing the clamp and advancing.
• See Methods Video S1, no backflow during the 1 min infusion period.

### Problem 4

Difficulty advancing catheter when releasing the clamp (Step 30).

### Potential solution

• Similar to problem 2. Make sure to move the catheter independently of the filament. Since the filament is occluding the MCAO, pushing the filament in further may induce tissue damage and hemorrhage. See Methods Video S1.

### Problem 5

Unable to initiate reperfusion (Step 31).

### Potential solution

• Advance the catheter first.
• Retract BOTH catheter and filament.
• Advance the catheter again to prevent it from popping out of the CCA.

## Resource availability

### Lead contact

Further information and requests should be directed to and will be fulfilled by the lead contact, Thomas Krieg (tk382@medschl.cam.ac.uk).

### Technical contact

For technical support, please contact Jordan J. Lee (jjl71@cam.ac.uk).

### Materials availability

This study did not generate new unique reagents.

### Data and code availability

Original data using this protocol are available at https://doi.org/10.1093/cvr/cvaf118.

## Acknowledgments

This work was supported by the 10.13039/501100000274British Heart Foundation (PG/23/11344 to T.K.), the 10.13039/100031707Medical Research Council UK (MC_UU_00028/4), a 10.13039/100010269Wellcome Trust Investigator award (220257/Z/20/Z) to M.P.M., and in part by the Wellcome Trust (226800/Z/22/Z) to Disease Model Core, Institute of Metabolic Science, 10.13039/501100000735University of Cambridge. Certain illustrations were created with BioRender.

## Author contributions

J.J.L. wrote the manuscript. M.P.M. and T.K. read, edited, and revised the manuscript.

## Declaration of interests

J.J.L., M.P.M., and T.K. have patents applications on targeting succinate metabolism in ischaemia/reperfusion injury. M.P.M. and T.K. are directors of Camoxis Therapeutics Ltd.
